# Development of early‐born γ‐Aminobutyric acid hub neurons in mouse hippocampus from embryogenesis to adulthood

**DOI:** 10.1002/cne.23961

**Published:** 2016-02-08

**Authors:** Vincent Villette, Philippe Guigue, Michel Aimé Picardo, Vitor Hugo Sousa, Erwan Leprince, Philippe Lachamp, Arnaud Malvache, Thomas Tressard, Rosa Cossart, Agnès Baude

**Affiliations:** ^1^ INSERM U901Marseille13009France; ^2^ Aix‐Marseille University, UMR 901Marseille13009France; ^3^ INMEDMarseille13009France

**Keywords:** long‐range projecting GABA neuron, inducible genetic fate mapping, operational hub, AB_10000240, AB_10550535, AB_477329, AB_2531897, AB_2302603, AB_2270299, AB_887864, nif‐0000–10294

## Abstract

Early‐born γ‐aminobutyric acid (GABA) neurons (EBGNs) are major components of the hippocampal circuit because at early postnatal stages they form a subpopulation of “hub cells” transiently supporting CA3 network synchronization (Picardo et al. [2011] Neuron 71:695–709). It is therefore essential to determine when these cells acquire the remarkable morphofunctional attributes supporting their network function and whether they develop into a specific subtype of interneuron into adulthood. Inducible genetic fate mapping conveniently allows for the labeling of EBGNs throughout their life. EBGNs were first analyzed during the perinatal week. We observed that EBGNs acquired mature characteristics at the time when the first synapse‐driven synchronous activities appeared in the form of giant depolarizing potentials. The fate of EBGNs was next analyzed in the adult hippocampus by using anatomical characterization. Adult EBGNs included a significant proportion of cells projecting selectively to the septum; in turn, EBGNs were targeted by septal and entorhinal inputs. In addition, most EBGNs were strongly targeted by cholinergic and monoaminergic terminals, suggesting significant subcortical innervation. Finally, we found that some EBGNs located in the septum or the entorhinal cortex also displayed a long‐range projection that we traced to the hippocampus. Therefore, this study shows that the maturation of the morphophysiological properties of EBGNs mirrors the evolution of early network dynamics, suggesting that both phenomena may be causally linked. We propose that a subpopulation of EBGNs forms into adulthood a scaffold of GABAergic projection neurons linking the hippocampus to distant structures. J. Comp. Neurol. 524:2440–2461, 2016. © 2016 Wiley Periodicals, Inc.

Cortical γ‐aminobutyric acid (GABA) neurons play an essential role in coordinating network oscillations during development and adulthood (Freund and Buzsáki, 1996; Klausberger et al., 2004; Bonifazi et al., 2009; Lapray et al., 2012; Royer et al., 2012; Varga et al., 2012). Within the adult hippocampus, GABA neurons form a diverse population (Freund and Buzsáki, 1996; Cossart et al., 2006; Ascoli et al., 2008; Klausberger and Somogyi, 2008). This diversity is determined partially by developmental programs. In particular the place and time of embryonic origin of cortical GABA neurons is a major determinant of their final phenotype (Anderson et al., 1997; Marin and Rubenstein, 2001; Butt et al., 2005; Fogarty et al., 2007; Wonders et al., 2008; Batista‐Brito and Fishell, 2009; Tricoire et al., 2011; Chittajallu et al., 2013; Matta et al., 2013; Kepecs and Fishell, 2014; Kessaris et al., 2014). Accordingly, we have shown that birth date critically determines GABA neuron network function because the cells generated the earliest during embryogenesis develop into a major functional subclass of superconnected “operational hub neurons” single‐handedly orchestrating network dynamics in the developing hippocampus at the end of the first postnatal week (Picardo et al., 2011). Given the potential role of hub neurons in activity‐dependent developmental processes and their strong contribution to hippocampal GABAergic connectivity, it is therefore essential to understand how these cells mature from embryonic to adult stages.

To this aim, we performed a full morpho‐physiological and molecular analysis of early‐born GABA neurons (EBGNs) by using a genetic fate mapping strategy. This approach is based on the fact that forebrain GABA neurons require the expression of the transcription factors *Dlx1* and *Dlx2* for their generation (Anderson et al., 1997; Wonders and Anderson, 2006). *Dlx1/2‐*expressing precursors were labeled with the transient activation of a *Dlx1/2* ^CreERTM^ driver line (Batista‐Brito et al., 2008) crossed with a Cre‐dependent EGFP reporter line, *RCE:LoxP* (Sousa et al., 2009), that allows for permanent labeling of GABA neurons by maternal tamoxifen administration at specific time points in gestation. We investigated the maturation of EBGNs during the perinatal period and examined their connectome in adulthood by using a compound experimental approach. The perinatal period was chosen because it is the time when population coherence emerges in the rodent hippocampus, following a precise sequence as shown in CA1 (Crepel et al., 2007).

We show that the morphofunctional features of EBGNs parallel the maturation of synchronous neuronal activity during the perinatal period. In adulthood, EBGNs are a subpopulation of long‐range neurons projecting to the septum, in agreement with their neurochemical identity and their displaying an axon running toward the fimbria (Picardo et al., 2011). We propose that there may be a broad developmental program by which GABA neurons that are generated the earliest are not simply “interneurons” but also are endowed with attributes supporting their developmental function. This study provides the template for future work studying the fate of EBGNs under pathological conditions.

## MATERIALS AND METHODS

### Inducible genetic fate mapping

All protocols were performed under the guidelines of the French National Ethic Committee for Sciences and Health report on “Ethical principles for animal experimentation,” in agreement with the European Community Directive 86/609/EEC under agreement #01413.03. All efforts were made to minimize pain and suffering and to reduce the number of animals used. We have fate mapped GABA neuron precursors expressing *Dlx1/2* by crossing *Dlx1/2* ^CreERTM/CreERTM^/*RCE:LoxP* ^+/+^ double‐homozygous males with wild‐type Swiss females. Swiss pregnant females were fed by means of a tube passed into the stomach (Fine Science Tools, Foster City, CA) with a tamoxifen solution (2 mg tamoxifen solution per 30 g body weight prepared at 10 mg/ml in corn oil, Sigma, St. Louis, MO) at embryonic days 7.5 after vaginal plug as reported by Picardo et al. (2011). Recombination of the reporter allele is achieved within 24 hours upon administration of tamoxifen, providing temporal precision in the labeling of cells expressing *Dlx1/2*.

### Assessing nonspecific labeling

Because “leaky” expression resulting in non‐tamoxifen‐dependent Cre‐mediated recombination had been previously reported (Madisen et al., 2010), we decided to test our mouse model, originally used to fate map the temporal origins of olfactory bulb GABA interneuron subtypes (Batista‐Brito et al., 2008). In living hippocampal slices (for in vitro imaging or electrophysiology) prepared from pups issued from non‐tamoxifen‐treated mothers, no EGFP labeling could ever be detected. However, when the EGFP signal was amplified through immunolabeling of transcardially paraformaldehyde‐perfused pups, some nonspecific labeling could be found. We have quantified the number of EGFP‐immunolabeled neurons per 70‐μm‐thick horizontal hippocampal sections from adult mice issued from tamoxifen‐treated (8.12 ± 1.03, n = 7 mice) and non‐tamoxifen‐treated mothers (0.9 ± 0.29, n = 3 mice). This indicated that approximately 11% of the EGFP‐immunolabeled EBGNs were in reality not necessarily early born because they resulted from leaky labeling in adult transgenic mice. Therefore, the quantitative data concerning the number of immunolabeled EGFP cells must be corrected by a factor 0.11. Caution should therefore be applied when using this *Dlx1/2* ^CreER^ driver mouse line with immunolabeling or a reporter mouse line stronger than RCE, such as Ai14 (Madisen et al., 2010). In addition, we observed that with time the fraction of unspecific labeling was augmented (not shown), which argues in favor of a constant renewal of the transgenic mouse colony.

### Slice preparation and calcium imaging

Horizontal hippocampal slices (350–380 μm thick, n = 61 slices) were prepared from tamoxifen‐treated *Dlx1/2* ^CreER/+^/*RCE:LoxP* ^+/–^ mouse pups of various ages from embryonic day 18.5 (E18.5) to postnatal day 7 (P7) as described previously (Picardo et al., 2011; Allene et al., 2012). Slices were incubated with 25 μl of a 1 mM Fura2‐AM solution (Invitrogen, Carlsbad, CA; in 100% DMSO) for 20–30 minutes. Imaging of the CA3 area was performed with a multibeam multiphoton pulsed laser scanning system (LaVision Biotech) coupled to a microscope as previously described (Bonifazi et al., 2009). Images were acquired through a CCD camera, which typically resulted in a time resolution of 50–150 msec per frame. Slices were imaged with a ×20, NA‐0.95 objective (Olympus). Imaging depth was on average 80 μm below the surface (range 50–100 μm). Analysis was performed with custom‐made software written in Matlab (Mathworks, Nattick, MA) as previously reported (Picardo et al., 2011). Active cells were considered to be neurons exhibiting at least one calcium event within the period of recording. Synchronous plateau assemblies activity (SPAs; Crepel et al., 2007) was quantified as the percentage of active cells exhibiting characteristic calcium plateaus (see Fig. 4C). Giant depolarizing potentials (GDPs; Ben‐Ari et al., 1989) were detected as the peaks of synchronous neuronal calcium events that exceeded 5% of active cells within the period of recordings.

### Electrophysiology

In total 97 EBGNs within the CA3 area were recorded in hippocampal slices of mice aged between E18.5 and P7 (86 cells) and from P25 mice (11 cells) for morphophysiological characterization. Neurons were held in current‐clamp using a patch‐clamp amplifier (HEKA, EPC10) in the whole‐cell configuration. Intracellular solution composition was (in mM): 130 K‐methyl‐SO_4_, 5 KCl, 5 NaCl, 10 HEPES, 2.5 Mg‐ATP, 0.3 GTP, and 0.5% neurobiotin. No correction for liquid junction potential was applied. The osmolarity was 265–275 mOsm, pH 7.3. Microelectrodes resistance was 4–8 MOhm. Uncompensated access resistance was monitored throughout the recordings. Values below 20 MOhm were considered acceptable, and the results were discarded if this changed by more than 20%. Whole‐cell measurements were filtered at 3 kHz by using a patch‐clamp amplifier. Recordings were digitized online (10 kHz) with an interface card to a personal computer and acquired in Axoscope 7.0 software. The resting potential (Vrest) was measured as the membrane potential baseline value obtained in current‐clamp mode in the absence of current injection. Frequency and amplitude of spontaneous excitatory postsynaptic potentials (sEPSPs) were detected and analyzed using MiniAnalysis software. Action potential threshold (Vthreshold) and amplitude and membrane capacitance (Cm) and resistance (Rm) were measured offline in Clampfit.

### Neurobiotin‐filled cell morphological analysis

In total 47 neurons from pups were recovered for morphological analysis. Slices were fixed overnight at 4°C in Antigenfix, rinsed in phosphate‐buffered saline (PBS) containing 0.3% Triton X‐100 (PBST) and incubated overnight at room temperature in Cy3‐streptavidin (1/1,000 in PBST; Jackson Immunoresearch, West Grove, PA). Morphological analysis of EBGNs has not been performed in adult mice, because, in contrast to the immature condition, axons, but also dendrites, were often truncated by the slicing. Post hoc analysis was performed using a confocal microscope. Stacks of optical sections were collected for neuronal reconstruction with a computer‐assisted system (Neurolucida; MicroBrightField, Williston, VT; RRID:nif‐0000‐10294). Morphological variables included dendritic and axonal lengths; somatic, dendritic, and axonal surfaces; and numbers of dendritic and axonal endings and nodes. Sholl analysis was performed to determine the distribution of the number of axonal intersections with circles of increasing radius (20‐μm steps) centered at the cell's soma.

### Tracing experiments

To map the possible long‐range projection targets of EBGNs, injections of the retrograde tracer fluorogold (10% in saline; Fluorochrome, LLC) were performed in various cortical and subcortical areas as previously described (Villette et al., 2012). All injections were performed in adult mice aged over 30 postnatal days. Single or multiple stereotaxic injections were performed. The constrained tissue was allowed to recover each time for 1 minute prior and after injection, so that it relaxes back to its original position and avoids injection backwash. Anteroposterior and mediolateral stereotaxic coordinates were calculated relative to bregma; dorsoventral stereotaxic coordinates were relative to the cortical surface (see Table 1; Paxinos and Franklin, 2001).

**Table 1:** TABLE 1A. Retrograde Tracing, Fluorogold Injections^1^

Targeted structures	Effective targeted structures	AP	ML	DV	Mice	Analyzed region
CA1	Whole dorsal hippocampus but mainly CA1	–2.05	1.5	–1.2	5	Contralateral hippocampus
CA3	CA3	–2.05	2.75	–2	5	Contralateral hippocampus
DG	Whole dorsal hippocampus but mainly DG	–2.05	1.2	–1.8	5	Contralateral hippocampus
Subiculum/retropspenial cortex	Subiculum and retrospenial cortex	–2.5/–3.4 –3.05/–3.8	± 0.3/ ± 0.7 ± 1.6/ ± 2.6	–0.6 –1.3/1.45	4	Whole hippocampus
Prelimbic area	Prelimbic area	+2	± 0.25	–1.5	5	Whole hippocampus
Entorhinal cortex	Perirhinal, entorhinal cortices, amygdaloid structures	–4.2	At ∼1.4 mm depth below the rhinal sinus with a 70–80° angle		6	Whole hippocampus
Medial septum	Septum and diagonal band of Broca	+0.8	0	–3.55	5	Whole hippocampus
CA1	Whole dorsal hippocampus but mainly CA1	–2.03	± 1.5	–1.7/–1.3	5	Septum Entorhinal cortex

TABLE 1B. Anterograde Tracing, *Phaseolus vulgaris* Injections^a^						
Targeted structures	Effective targeted structures	AP	ML	DV	Mice	Analyzed region
Entorhinal cortex	Entorhinal cortex	–3.5	+3.9	–2.9	4	Contralateral hippocampus
Medial septum	Septum and diagonal band of Broca	+0.8	0	–3.55	4	Contralateral hippocampus
CA1	Dorsal hippocampus	–2.05	± 1.5	–1.2	5	Septum, entorhinal cortex

a AP, anterioposterior: ML, mediolateral; DV, dorsoventral. All coordinates are in millimeters; ± for ML signifies bilateral injections.

For retrograde and anterograde tracer injections, animals were anesthetized with a ketamine (100 mg/kg) and xylazine (10 mg/kg) solution (i.p.). In total 70 adult male mice were injected in several hippocampal target structures (Jinno et al., 2007), including the medial septum, contralateral hippocampus (CA1, CA3 and dentate gyrus), prefrontal cortex (mainly prelimbic, infralimbic and cingulate cortices), entorhinal cortex, subiculum, and retrosplenial cortex. After verification of the accuracy of tracer injection sites, final results were from a total of 53 mice (Table 1). For easier access to the medial septum, the medial sinus was disconnected before pipette insertion (Villette et al., 2012). Because of the spread of the fluorogold solution, injections in the dorsal subiculum and retrosplenial cortex were designed and considered as a single group. To avoid labeling of parietal cortices such as visual and auditory cortices during the descent of the pipette, a tangential stereotaxic approach was used for injections in the entorhinal cortex. To test whether septal and/or entorhinal EBGNs projected to the hippocampus, fluorogold was injected bilaterally in the hippocampus. All fluorogold injections were performed using a nanofil (35 G; WPI, Sarasota, FL) coupled to a nanosyringe (10 μl; WPI). A volume of 50 nl or 100 nl was pressure injected at a rate of 50 nl/minute, except for entorhinal cortex injections that used 10–12 psi for 10 msec and a Picospritzer III (General Valve Corporation). Septal and entorhinal inputs to hippocampal EBGNs were examined with a single iontophoretic injection of the anterograde tracer *Phaseolus vulgaris* type L (PhaV rhodamine labeled; Vector Laboratories, Burlingame, CA; see Table 1). Similarly, hippocampal afferents to septal EBGNs were examined by using unilateral single iontophoretic injections of PhaV in the hippocampus. A 5‐μA positive current was applied between a glass pipette containing the PhaV solution (10% in saline) and a reference electrode during 7‐second on–off cycles.

Just after tracer injections, animals were rehydrated with a subcutaneous injection of saline, placed back in their home cage, and checked daily for full recovery. Mice were anesthetized about 1 week after the injections and perfused with paraformaldehyde. Brain sections were processed for multiple labeling as described below.

### Immunocytochemistry

Adult animals were anesthetized with a ketamine (250 mg/kg) and xylazine (25 mg/kg) solution (i.p.) and transcardially perfused with 4% paraformaldehyde in PBS (1 ml/g). E18.5 embryos were excised after deep anesthesia of pregnant mothers; embryos' brains were extracted and immersed in fixative. After anesthesia in isoflurane, P0–P2 brains were extracted and immersed in fixative, whereas P3–P8 pups were perfused with paraformaldehyde before brain extraction. Brains were postfixed overnight, washed in PBS, then placed in 30% sucrose in PBS before freezing in liquid nitrogen and kept at –20°C for long‐term storage.

Coronal or horizontal brain sections were prepared and processed for immunohistochemistry as described previously (Picardo et al., 2011). Various primary antibodies were used (Table 2). Secondary antibodies (dilution 1:500) used were donkey anti‐chicken DyLight 488, donkey anti‐rabbit DyLight 549, donkey anti‐guinea pig Cy3, donkey anti‐mouse DyLight 549, and donkey anti‐goat DyLight 649 (all from Jackson Immunoresearch, West Grove, PA).

**Table 2:** Antibodies Used To Label EBGNs in Mouse Forebrain

Antigen	Immunogen	Source, host species, catalog No., clone or lot No., RRID	Concentration used
Calbindin	Purified human calbindin D28k (3–251 aa)	Synaptic Systems, polyclonal guinea pig, 214 004, AB_10550535	1:1,000
EGFP	Recombinant green fluorescent protein	Aves Labs., polyclonal chicken, GFP‐1020, 0316FP11, AB_10000240	1:1,000
KCC2	Rat KCC2 (929–1045 aa)	Generous gift from Dr. C. Rivera, polyclonal rabbit, Ludwig et al. (2003)	1:3,000
mGluR1α	Mouse mGluR1α (945–1127 aa)	Frontier Institute, polyclonal guinea pig, mGluR1a‐GP‐Af660‐1, AB_2531897	1:1,000
Parvalbumin	Purified frog muscle parvalbumin	Sigma‐Aldrich, monoclonal mouse, Parv‐19, P3088, AB_477329	1:1,000
Somatostatin	Purified peptide from human somatostatin C‐terminus	Santa Cruz Biotechnology, polyclonal goat, sc‐7819, AB_2302603	1:2,000
VAChT	Rat VAChT (475–530 aa)	Synaptic Systems, polyclonal rabbit, 139 103, AB_887864	1:500
VMaT2	Rat VMaT2 (496–515 aa)	Synaptic Systems, polyclonal rabbit, 138 302, AB_2270299	1:500

### Antibody characterization

The calbindin guinea pig antiserum recognized a single band at 28 kDa in Western blot preparations from rat brain homogenate and stained a pattern of cellular morphology and distribution in the mouse hippocampus (Puighermanal et al., 2015; Table 2).

Specificity of the EGFP chicken antiserum has been previously analyzed by Western blot with transgenic mice expressing the GFP gene product; Western blot analysis produced a single band of 28 kDa (manufacturer's technical information). In addition, no staining was detected on brain sections from wild‐type mice (Zhao et al., 2008; Table 2).

The KCC2 rabbit antiserum was a generous gift from Dr. Claudio Rivera. On Western blot of whole‐brain homogenates from adult mice, the KCC2 antibody recognized two bands at 150 and 250 kDa corresponding to the glycosylated form and to an aggregated form of KCC2, respectively; in addition, no staining was observed with KCC2 knockout mice (Ludwig et al., 2003). Moreover, labeling distribution was similar to that previously published (Tyzio et al., 2014; Table 2).

The mGlur1α guinea pig antiserum detected a single protein band at 145 kDa in Western blot of mouse brain preparation and stained soma and dendrites of cortical neurons as previously observed (Picardo et al., 2011; Table 2).

The parvalbumin mouse antibody recognized a 12‐kDa band from human, bovine, pig, canine, feline, rabbit, rat, and fish tissues (manufacturer's technical information). The localization and morphology of hippocampal neurons stained with this antiserum were identical to those in previous reports (Freund and Buszaki, 1996; Picardo et al., 2011; Table 2).

The somatostatin goat antiserum identified a 17‐kDa human recombinant somatostatin fusion protein by Western blotting (manufacturer's technical information), and no labeling was observed in tissue from knockout mice (Lepousez et al., 2010; Table 2).

The vesicular acethylcholine transporter (VAChT) rabbit antibody recognizes a strong band at ∼50 kDa and weaker bands at 67 kDa and 80 kDa on Western blots of mouse brain preparations (Fortune and Lurie, 2009). In addition, specificity has been verified on knockout mice (manufacturer's technical information; Table 2).

The vesicular acethylcholine transporter isoform 2 (VMaT2) rabbit antibody recognized proteins with the expected molecular weight in immunoblots, with a strong band at ∼55 kDa on Western blots of mouse brain preparations (Fortune and Lurie, 2009). Moreover, the pattern of immunolabeling in the hippocampus was similar to that previously reported (Amaral and Lavenex, 2007; Table 2).

### Image acquisition and analysis

Images were obtained with a Zeiss AxioImager Z2 microscope coupled to a camera (Zeiss AxioCam MR3) with an HBO lamp associated with (470/40, 525/50), (545/25, 605/70) filter cubes. Confocal images were obtained with a Leica TCS SP5‐X equipped with emission spectral detection and a tunable laser providing excitation range from 470 to 670 nm. Some sections obtained from adult mice were processed for EGFP immunoperoxidase for electron microscopy (Dufour et al., 2010). Ultrathin sections (80 nm) were obtained with an ultramicrotome (Ultracut E; Reichert‐Jung) and examined on a Zeiss EM 912 electron microscope equipped with a digital camera (Advanced Microscopy Techniques, Danvers, MA).

To evaluate the distribution of labeled neurons (EGFP positive, immunohistochemically labeled, retrogradely labeled, etc.) along the rostrocaudal axis of the adult hippocampus, cells were manually counted on coronal sections according to three levels as defined from the atlas of Paxinos and Franklin (2001): 1) rostral level: from bregma –0.94 mm to bregma –1.70 mm; 2) intermediate level: from bregma –1,82 mm to bregma –2,80 mm; and 3) caudal level: from bregma –2.92 mm to bregma –3,88 mm. All results are given as percentages of the total number of EGFP‐expressing EBGNs or as means of percentages ± SEM.

## RESULTS

Before presenting our results, we believe it is important to stress that caution should be taken when considering tamoxifen‐dependent activation of the Cre recombinase. Indeed, tamoxifen‐independent labeling could be observed restricted to the GABA neuron population, which accounted for approximately 10% of the overall labeled population when using the RCE reporter line (see Materials and Methods). Although this constitutive leak was not visible on living hippocampal slices prepared for electrophysiology, it could be considerably amplified by immunolabeling or through the use of a stronger reporter as Ai14. When working with small numbers iof EBGNs, such leaky reporter expression could become critical.

### Morphophysiological properties of EBGNs in the developing CA3

**Figure 1:**
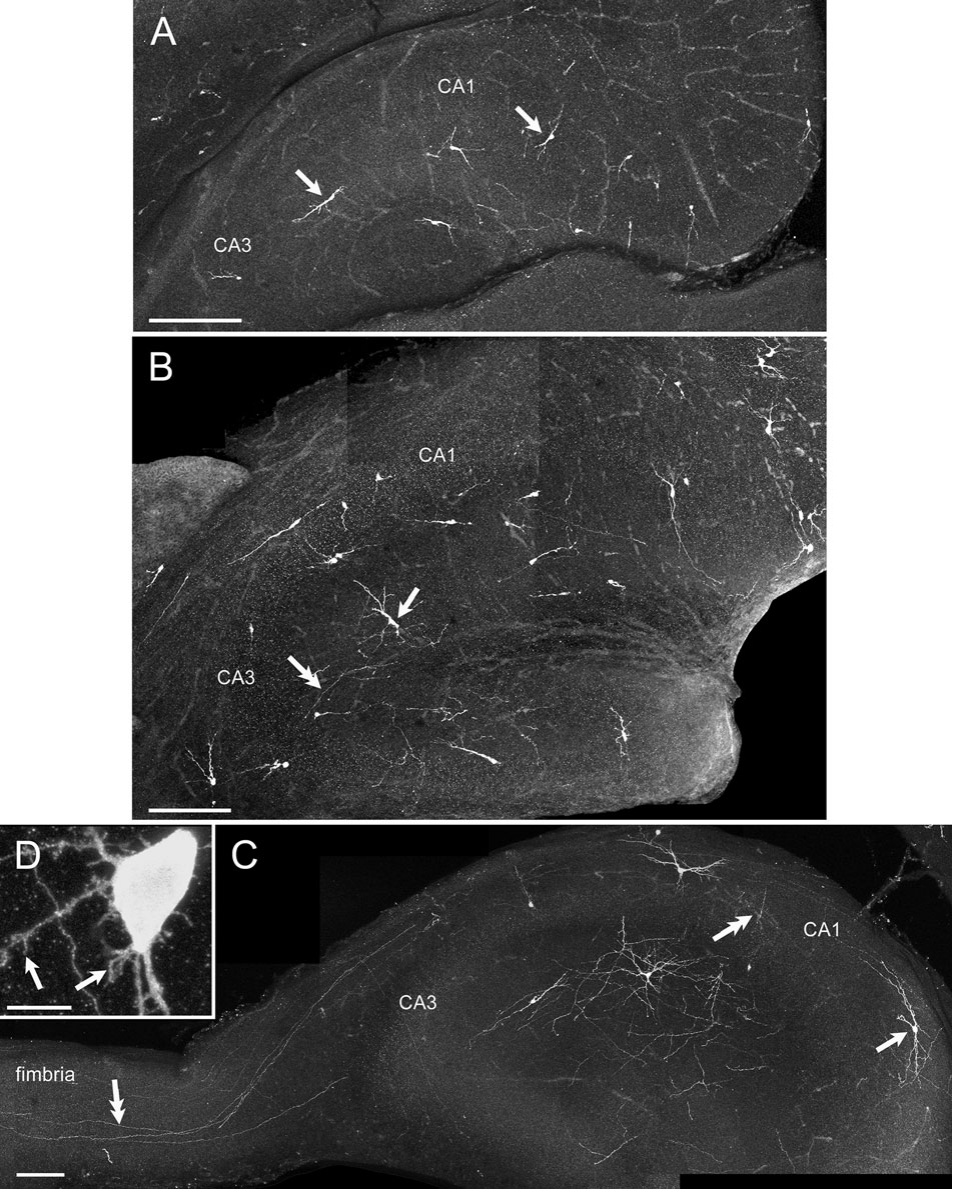
Morphological features of EBGNs in developing hippocampus. Sparse fate‐mapped neurons are visible in the developing hippocampus in a *Dlx1/2* ^CreERTM^/*RCE:LoxP* mouse tamoxifen treated at E7.5. **A:** E18.5; most of the EBGNs have a fusiform soma with a poorly developed dendritic tree (arrows). **B:** P0; The dendritic (single arrow) and axonal (double arrow) arborizations of EBGNs increase and become more complicated. **C:** P3; EBGNs show a conspicuous development of the dendritic (single arrow) and axonal (double arrow) arborizations; several axons are seen running through the fimbria (double arrow). **D:** Filopodia (single arrows) are visible at the soma and dendrites of an EBGN at P3. Scale bars = 200 μm in A–C; 10 μm in D.

During the first postnatal week, EBGNs were found in all layers of the hippocampus on sections obtained from paraformaldehyde‐fixed brains and immunolabeled for EGFP (Fig. 1C). Their morphology developed from fusiform with few processes at E18.5 to displaying heavily branched dendrites and axons often running over long distances at P3. The EBGNs presented filopodia during the perinatal period (Fig. 1). In total 95 cells were analyzed, and 18/27 cells (67%) from three pups, 46/53 cells (87%) from five pups, and 13/15 cells (87%) from three pups exhibited filopodia at E18.5, P1, and P3, respectively. In contrast, none of the 49 EBGNs from three pups at P7 had filopodia. Given that filopodia were previously designated among the characteristic morphological features of GABA neurons involved in SPAs (Allene et al., 2012), this could indicate a change in spontaneous firing of EBGNs toward the end of the first postnatal week. To test for this issue, EGFP‐expressing cells were targeted for electrophysiological recordings and neurobiotin filling in the CA3 region of hippocampal slices from E18.5 until the end of the first postnatal week (P7). In total 86 EBGNs from 36 pups were recorded in current‐clamp mode and pooled into three successive age groups (Table 3), perinatal (E18.5–P1, 27 cells from 12 pups), midweek (P2–4, 21 cells from 10 pups), and end‐of‐week (P5–P7, 38 cells from 14 pups) periods. In addition, 11 cells were recorded from six adult animals (P25–P45). In agreement with the analysis of immunolabeled EGBNs, we found that some EBGNs displayed spontaneous and evoked firing patterns that indicated their involvement in SPAs until the midweek period, but, toward the end of the postnatal week, EBGNs displayed evoked firing pattern diversity and were all involved in GDPs (Table 3). The threshold for action potential generation was also significantly more hyperpolarized by almost 5 mV at P5–P7 than earlier but was similar to adult values, further supporting a functional maturation of EBGNs (Table 3). We observed a significant increase in the frequency of spontaneous EPSPs (sEPSPs) received by these neurons at resting membrane potential (Table 3). We also analyzed the maturation of the subthreshold properties of EBGNs and observed a significant decrease in membrane input resistance paralleled by an increase in membrane capacitance and a hyperpolarization of resting membrane potential (Table 3). Altogether, these changes clearly reflect the dramatic maturation of EBGN physiological properties between birth and P7 in CA3. Except from the rate and amplitude of sEPSPs, which most likely reflect continuous network maturation, most electrophysiological parameters describing EBGNs reached their adult values as early as P5–P7 (Table 3 ; see also Fig. 5). It is important to note that firing patterns are distributed differently into adulthood (Table 3). Because we did not use blockers for synaptic transmission during our recordings, these differences may also be explained by the progressive network maturation.

**Table 3:** Perinatal Development of the Morphofunctional Properties of EBGNs^a^

	Perinatal period (E18.5–P1)	Midweek period (P2–P4)	End‐of‐week period (P5–P7)	Adulthood (P25–P45)
Electrophysiological properties				
Number of cells	27	21	38	11
Rm (MOhms)****	1,624 ± 168	1,027 ± 74.7	698.3 ± 60.7	742.9 ± 88.75
Cm (pF)**	40 ± 3.2	64.3 ± 8	81.45 ± 10	69.11 ± 11.56
Vthresh (mV)***	–34 ± 1.2	–35.3 ± 2.2	–40.7 ± 1.03	–38.18 ± 1.85
AP amplitude (mV)**	34 ± 2.7	38.55 ± 2.84	44.74 ± 1.78	47.15 ± 3.49
AP duration (msec)***	3.25 ± 0.24	2.61 ± 0.23	2.44 ± 0.21	1.81 ± 0.34
Vrest (mV)	–37.25 ± 2.45	–43 ± 2.41	–44.7 ± 2	–40.10 ± 2.52
sEPSP frequency (Hz)****	0.81 ± 0.17	1.35 ± 0.39	2.93 ± 0.53	10.49 ± 1.35
sEPSP amplitude (mV)****	3.94 ± 0.29	3.26 ± 0.47	3.21 ± 0.23	1.39 ± 0.26
Firing pattern				
Number of cells	20	15	17	11
Percentage of adaptating spiking cells	90	67	18	64
Percentage of mixed spiking cells	5	13	6	27
Percentage of regular spiking cells	5	20	76	9
Percentage of cell with GDPs	40	80	100	0
Morphology				
Number of cells	15	9	23	n.a.
Cell body area (μm^2^)***	115.6 ± 15.8	135.2 ± 14.6	277.7 ± 25	—
Axon				
Nodes number, ns	61.2 ± 13.5	113.9 ± 93.3	174.6 ± 44.8	—
Ends number, ns	64.6 ± 14	121.1 ± 98.8	183 ± 46.5	—
Length (mm)***	1.78 ± 0.3	2.83 ± 0.67	8.10 ± 1.48	—
Dendrites				
Nodes number*	47.2 ± 12.1	79.4 ± 14.548	50.4 ± 60.5	—
Ends number*	55.3 ± 13.2	92 ± 14.7	60.5 ± 15.79	—
Length (mm), ns	1.16 ± 0.03	1.30 ± 0.21	1.80 ± 0.27	—
KCC2 expression				
Number of cells	36	104	51	18
Percentage of KCC2‐expressing cells**	0	8 ± 4	54 ± 9	68 ± 17

a ns, No statistical difference; *P* values as given by Kruskal and Wallis test. All data are given as mean ± SEM. AP, action potential; Cm, membrane capacitance; GDPs, giant depolarizing potential; Rm, membrane resistance; sEPSP, spontaneous excitatory postsynaptic potential; Vthresh, action potential threshold; Vrest, resting potential. n.a., not applicable.

*P < 0.05.

**P < 0.01.

***P < 0.0005.

****P < 0.0001.

**Figure 2:**
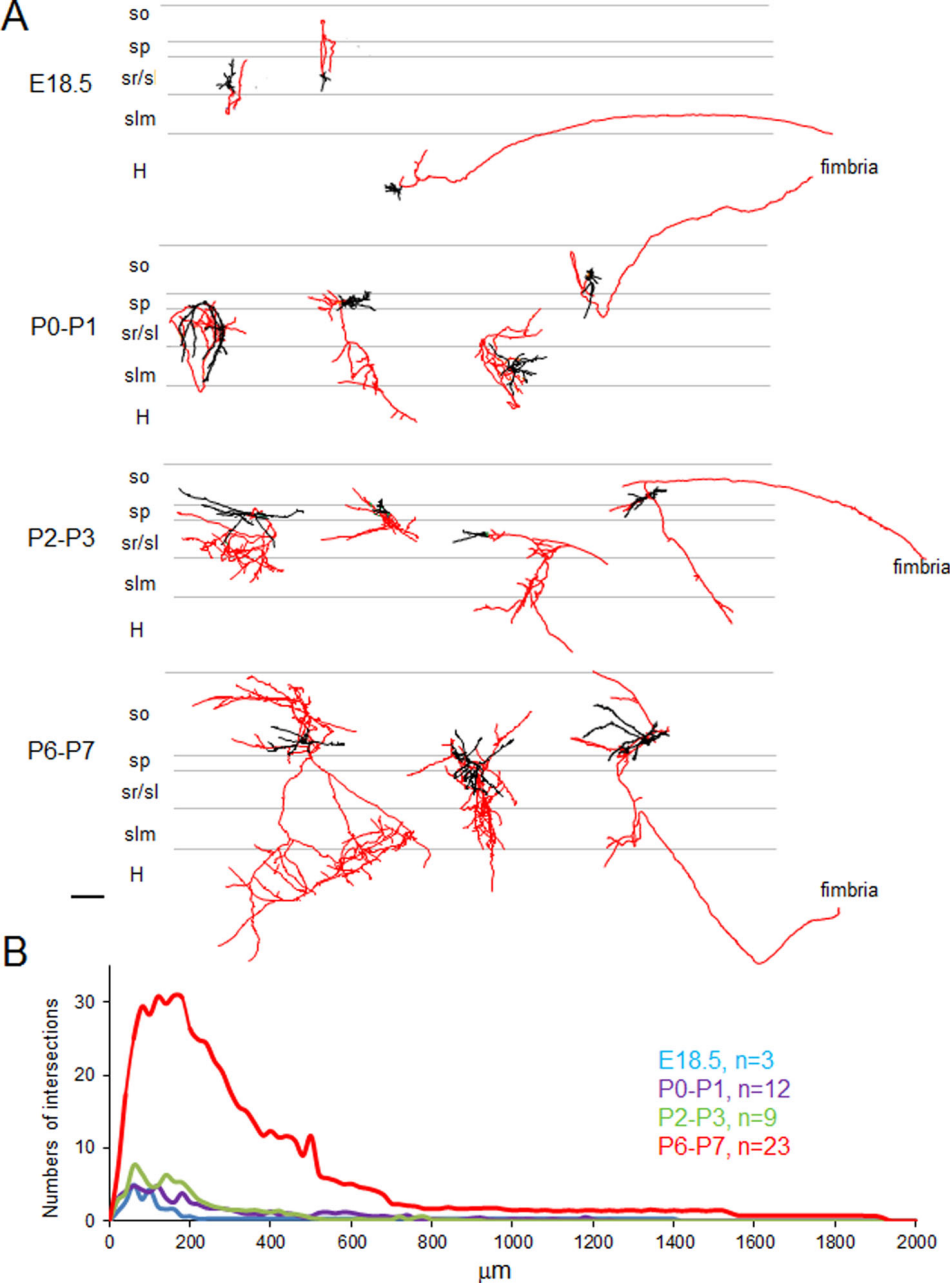
Development of the morphometric properties of EBGNs in CA3 during the perinatal period. **A:** Composite drawings of neurobiotin‐filled interneurons reconstructed with the Neurolucida workstation. The somatodendritic domains of EBGNs are represented in black. The axons are in red; note that some axons are leaving the hippocampus via the fimbria as early as E18.5. H, hilus; sl, stratum lucidum; slm, stratum lacunosum moleculare; sp, stratum pyramidale; so, stratum oriens; sr, stratum radiatum. **B:** Normalized distribution graphs of the fraction of axonal intersections with concentric circles of increasing radius (Sholl analysis, 20‐μm steps) centered at the soma for the cell populations described in A. Scale bar = 100 μm.

We next examined whether these changes were followed by a comparable evolution of morphological properties. To this aim, in total 47 EBGNs were reconstructed for morphometric analysis (Fig. 2, Table 3) from two embryos at E18.5 (three cells), six pups at P0–P1 (12 cells), six pups at P2–P3 (nine cells), and 14 pups at P6–P7 (23 cells). They were distributed in all CA3 subfields. Different types of morphologies could be recovered (Fig. 2A). At E18.5, cells were mostly fusiform, with an elongated soma and poorly developed dendrites. Then, dendritic trees became more elaborate and displayed an increase in their number of nodes and length (Table 3). Cells could be bipolar, with horizontally or vertically elongated dendrites, or multipolar. Still, the most significant change concerned the axonal compartment; axonal length increased by a factor of 4 during the second half of the first postnatal week. The axonal coverage of EBGNs often crossed subfield boundaries as soon as E18.5 and increased throughout the examined period. A measure of their extended axonal coverage is the distribution of the number of intersections that their axon makes with concentric circles of increasing radius centered at the soma (Sholl analysis). The axonal coverage extended mildly between E18.5 and P3 but then abruptly increased between P3 and P7 (Fig. 2B). Importantly, at all examined ages, axons emanating from EBGNs were seen running in the fimbria (Fig. 2A; see also Fig. 1).

**Figure 3:**
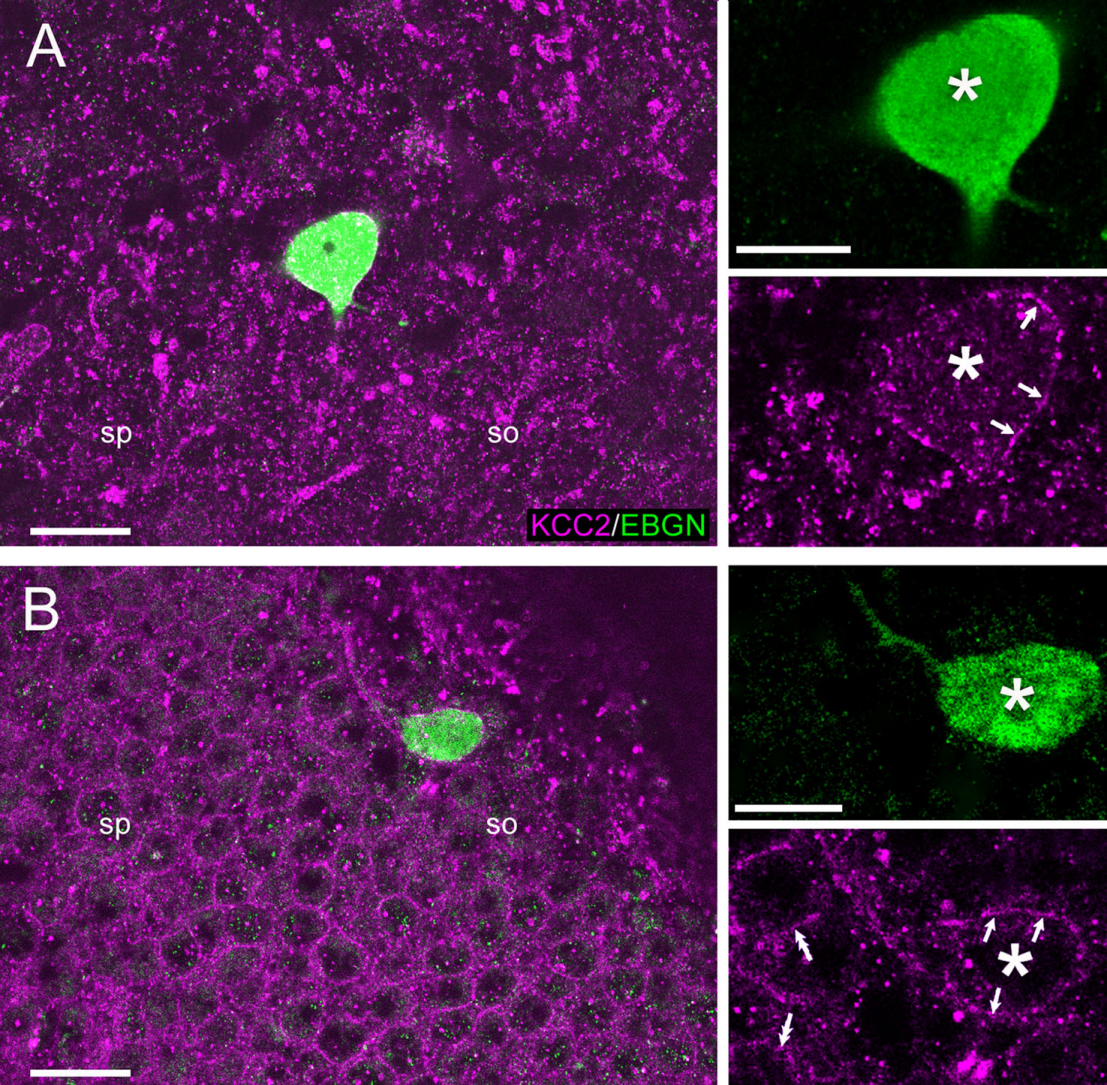
Perisomatic KCC2 expression in hippocampal EBGNs. Immunolabeling for KCC2 is visible along the membrane (single arrows) of EBGNs (asterisks) in CA1 stratum oriens (so) at P3 (**A**) and in adulthood (**B**; P45). Note the presence of similar labeling in neighboring neurons (double arrows in B) only at P45. sp, Stratum pyramidale. Scale bars = 20 μm at left; 10 μm at right.

Finally, we decided to study the expression of the chloride extruder pump KCC2 on EBGNs, because this is a molecular marker that represents a good proxy for functional neuronal maturation. Indeed, most mature CNS neurons exhibit high levels of KCC2 expression, whereas low expression is detected in immature neurons (for review see Chamma et al., 2012; Kaila et al., 2014; Medina et al., 2014). In addition, in mature neurons, KCC2 immunolabeling exhibits a perisomatic distribution (Baldi et al., 2010; Tyzio et al., 2014). Therefore, we examined the perisomatic expression of KCC2 in EBGNs (Fig. 3A,B) as a biomarker of neuronal maturation during the first postnatal week. In total 209 EBGNs were examined from three pups at P0 (36 cells), four pups at P2 (36 cells), four pups at P3 (68 cells), three pups at P7 (51 cells), and three adult mice at P45 (18 cells).The proportion of EBGNs showing perisomatic labeling increased with time (Table 3) and reached almost adult levels (68% ± 17%) at P7 (54% ± 8%).

Therefore, the morphophysiological properties of EBGNs develop at a fast pace during the first postnatal week, with an acceleration of axonal growth and an increase in the frequency of spontaneous synaptic inputs at about P3. This indicates that these cells that function as hub neurons at P7 increase their connectivity range at about P3. We therefore asked whether this could indicate the time at which the first synapse‐driven correlated activities emerged, in the form of GDPs (Ben‐Ari et al., 1989).

### Early postnatal development of EBGNs mirrors the emergence of GDPs

**Figure 4:**
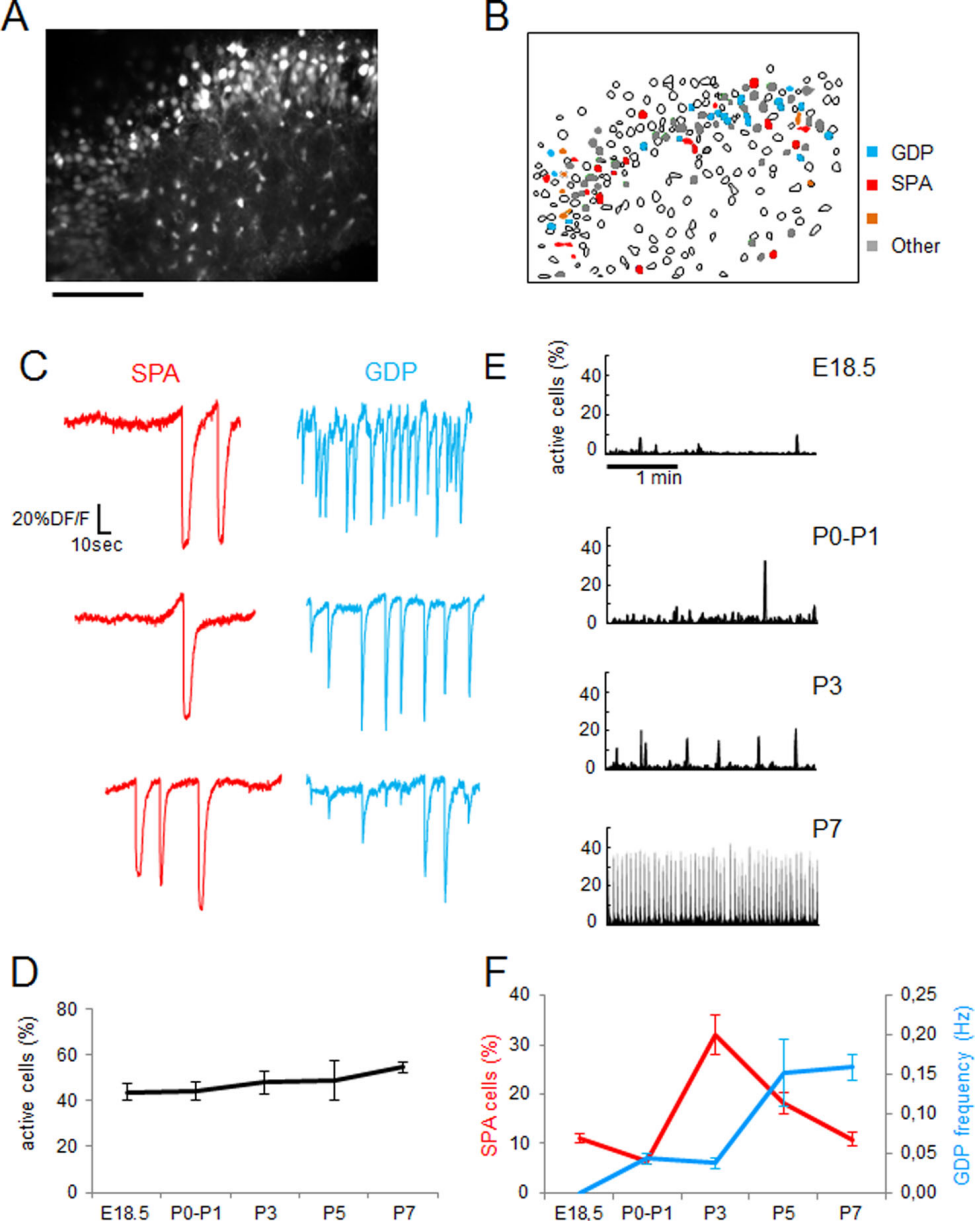
Maturation of correlated neuronal activities in the CA3 region during the perinatal period. **A:** Two‐photon calcium fluorescence image of the CA3 region of a mouse hippocampal slice at P3. **B:** Automatically detected contours of cells from the calcium fluorescence image (A1); silent cells (open contours); active cells (solid contours) could display SPAs (red), GDPs (blue), or both (orange). **C:** Calcium fluorescence traces as a function of time of six active cells from the movie illustrated in A showing calcium plateau characteristics of SPAs (red) or fast calcium transients associated with GDPs (blue). **D:** The percentage of active cells remains constant throughout the investigated ages. **E:** Representative histograms showing the percentage of imaged cells detected as being active at each movie frame from E18.5 to P7 (time resolution 100 msec). There is a clear increase in the frequency of GDPs (peaks of synchrony; threshold is 5% of coactive cells) and in their amplitude (i.e. percentage of coactive cells at peaks of synchrony). **F:** Graph indicates the fraction of SPA cells (red) as well as the frequency of GDPs (blue) for five successive age groups. Error bars indicate SEM. All data were obtained from n = 9 movies/n = 6 mice at E18.5; 6/2 at P0–P1; 13/5 at P3; 4/2 at P5; 26/12 at P7.

With this aim, we imaged the evolution of spontaneous neuronal activity in the CA3 region by using two‐photon calcium imaging. We had previously described the emergence of coordinated activities, but in the developing CA1 (Crepel et al., 2007). In total 58 calcium movies were analyzed: nine movies at E18.5 (six embryos), six movies at P0–P1 (three pups), 13 movies at P3 (five pups), four movies at P5 (two pups), and 26 movies at P7 (12 pups). Imaging the CA3 region during perinatal period revealed various calcium dynamics (Fig. 4A–C); active cells could be involved in SPAs, GDPs, both, or neither. Although the percentage of active cells remained constant (Fig. 4D), the contribution of different forms of coordinated activities to network dynamics evolved with time (Fig. 4E). At embryonic stages (E18.5), only SPA activity was observed, although in <10% of active cells (Fig. 4F). At birth (P0–P1) and later (P3), 10–30% of active cells produced SPAs; in parallel GDPs appeared, but their frequency remained low (<0.05 Hz; Fig. 4F). Then, SPAs were progressively replaced by GDPs that became the predominant form of synchronous activity toward the end of the first postnatal week (Fig. 4E,F). The frequency of GDPs increased twofold from the perinatal period to the midweek period, then by a factor of 4 between the midweek period and the end‐of‐week period (Table 4).

**Table 4:** Network Dynamics of the Developing CA3 Region^a^

	Perinatal period (E18–P1)	Midweek period (P2–P4)	End‐of‐week period (P5–P7)
Number of calcium movies	15	13	30
Percentage of active cells, ns	53 ± 4	48 ± 5	54 ± 2
Percentage of SPA cells****	9 ± 1	33 ± 4	12 ± 1
GDPs frequency (Hz)****	0.02 ± 0.004	0.04 ± 0.01	0.16 ± 0.01

a ns, No statistical difference; *P* values as given by Kruskal and Wallis test. All data are given as mean ± SEM.

****P < 0.0001.

**Figure 5:**
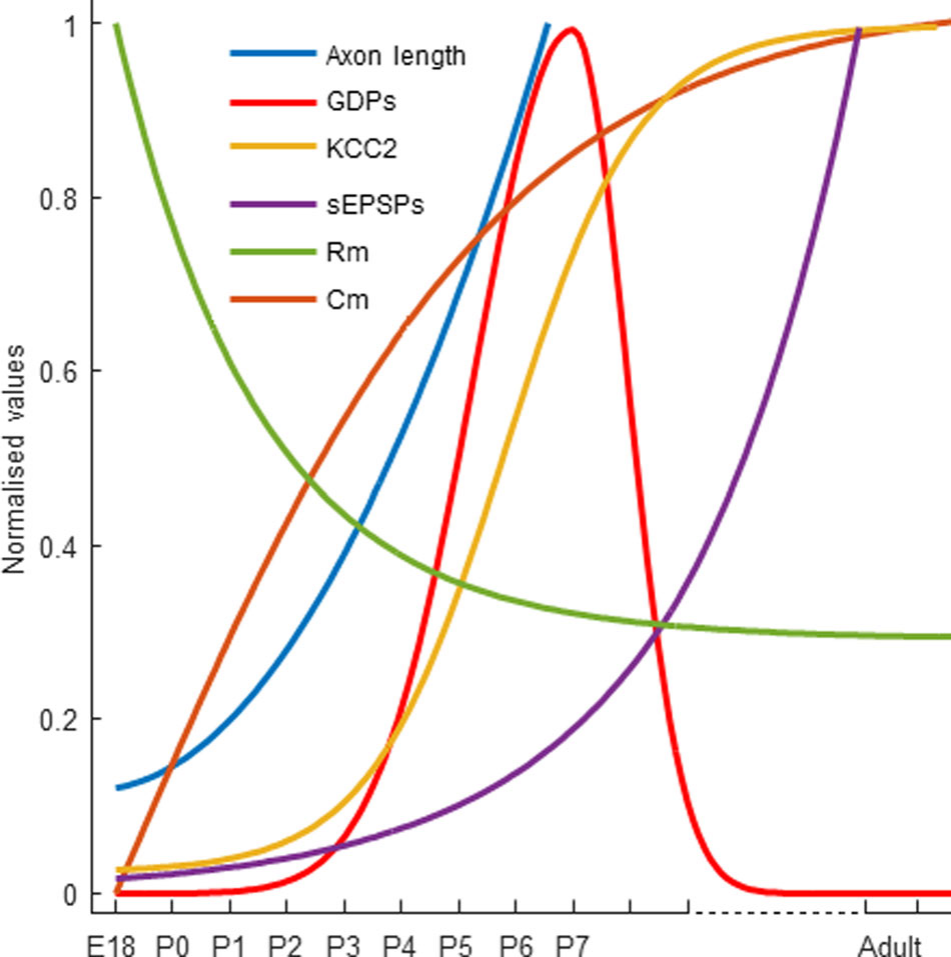
Parallel evolution of the properties of EBGN and and the rate of GDPs from embryogenesis to adulthood. Previously reported data were normalized and fitted using ad hoc functions (least square algorithm). Hyperbolic tangent for KCC2 (r^2^ = 0.99) and for Cm (membrane capacitance; r^2^ = 0,84), Gaussian (r^2^ = 0.97) from E18 until P7 then extrapolated for later ages for GDPs, exponential for sEPSPs (r^2^ = 0.99) and for Rm (membrane resistance, r^2^ = 0.98). Axonal length was measured only during the perinatal period. It was fitted using a parabola (r^2^ = 0.86).

We conclude that the morphophysiological development of EBGNs mirrors the maturation of neuronal network activities during the perinatal period. Synapse‐driven synchronous activity (GDPs) emerged at about P3 to become predominant at P7, in parallel to the increase in axonal growth of EBGNs (Fig. 5). This supports the idea that the morphophysiological maturation of EBGNs to hub neurons may be causally linked to the evolution of early network dynamics.

### Hippocampal EBGNs project to the septum in adult mice

**Figure 6:**
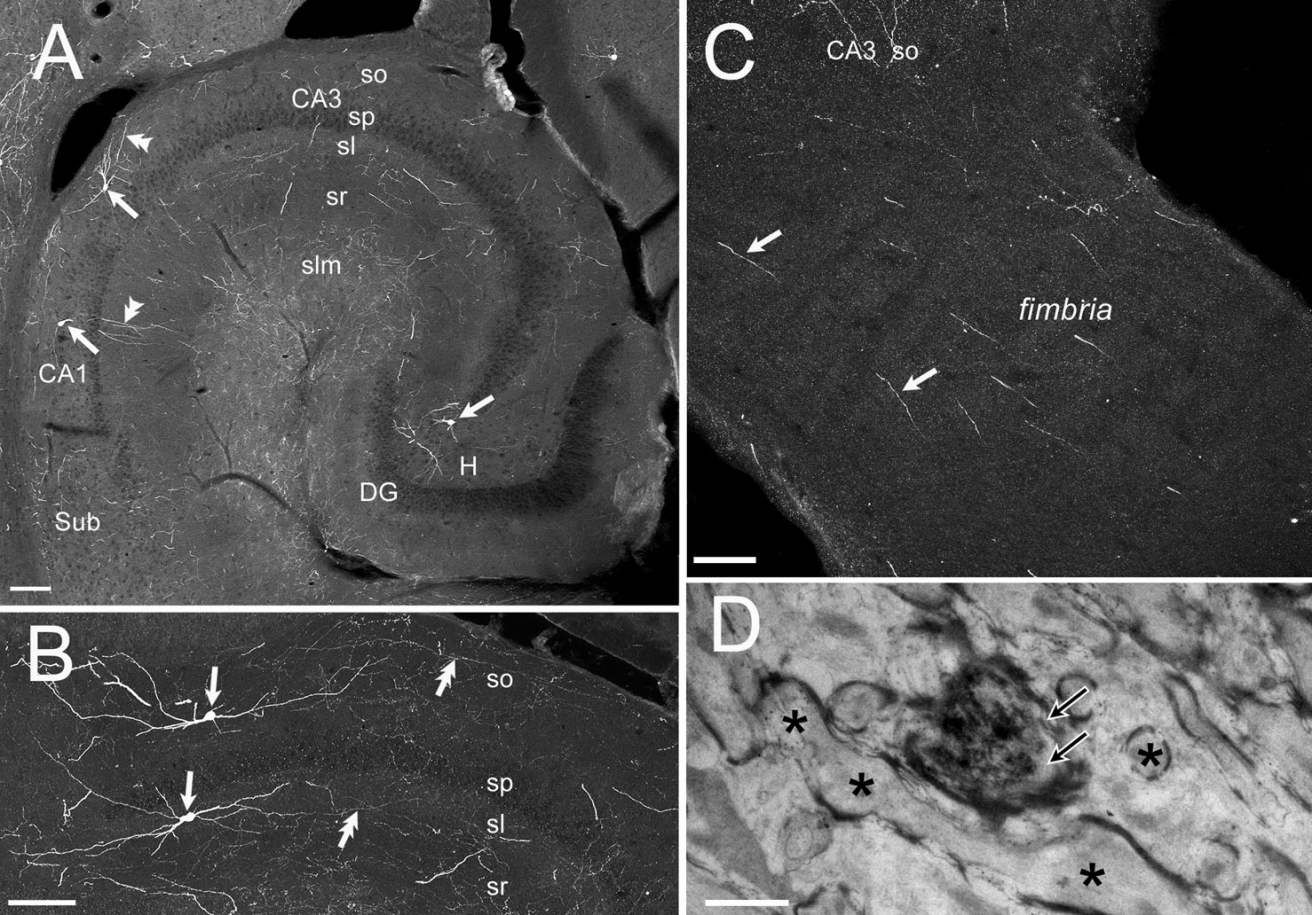
Distribution of EBGNs in the adult mouse hippocampus. Sparse EGBNs are visible in CA1, CA3 and the hilus (H) of the dentate gyrus (arrows in A,B).They have horizontally or vertically oriented dendrites (double arrowheads in A). Their axons are found in all sublayers (e.g., double arrows in B). Numerous EGFP‐labeled EGBNs' axons are observed in the fimbria (arrows in C). Electron microscopic view of the fimbria (D) showing a myelinated EBGN axon (arrows in D) following EGFP immunoperoxydase treatment. This axon is running along with unlabeled myelinated axons (asterisks). DG, dentate gyrus; H, hilus; sl, stratum lucidum; slm, stratum lacunosum moleculare; sp, stratum pyramidale; so, stratum oriens; sr, stratum radiatum; Sub, subiculum. Scale bars = 100 μm in A,B; 25 μm in C, 10 μm in D.

EBGNs were distributed throughout the septotemporal axis of the adult hippocampus in all sublayers. Neurons could be bipolar extending horizontally or vertically or could be multipolar (Fig. 6A,B). They were mostly aspiny, except in the stratum lucidum of CA3, where occasional spiny EBGNs could be detected. In addition, relatively large and spiny neurons were observed in the hilus. Similarly to what we had observed during development, numerous EGFP labeled axons were found in the fimbria (Fig. 6C); they were myelinated as evidenced by electron microscopy (Fig. 6D). The latter observation suggested that hippocampal EBGNs, at least some of them, might display a long‐range extrahippocampal projection in adulthood. To test this hypothesis, retrograde tracing experiments were performed.

**Figure 7:**
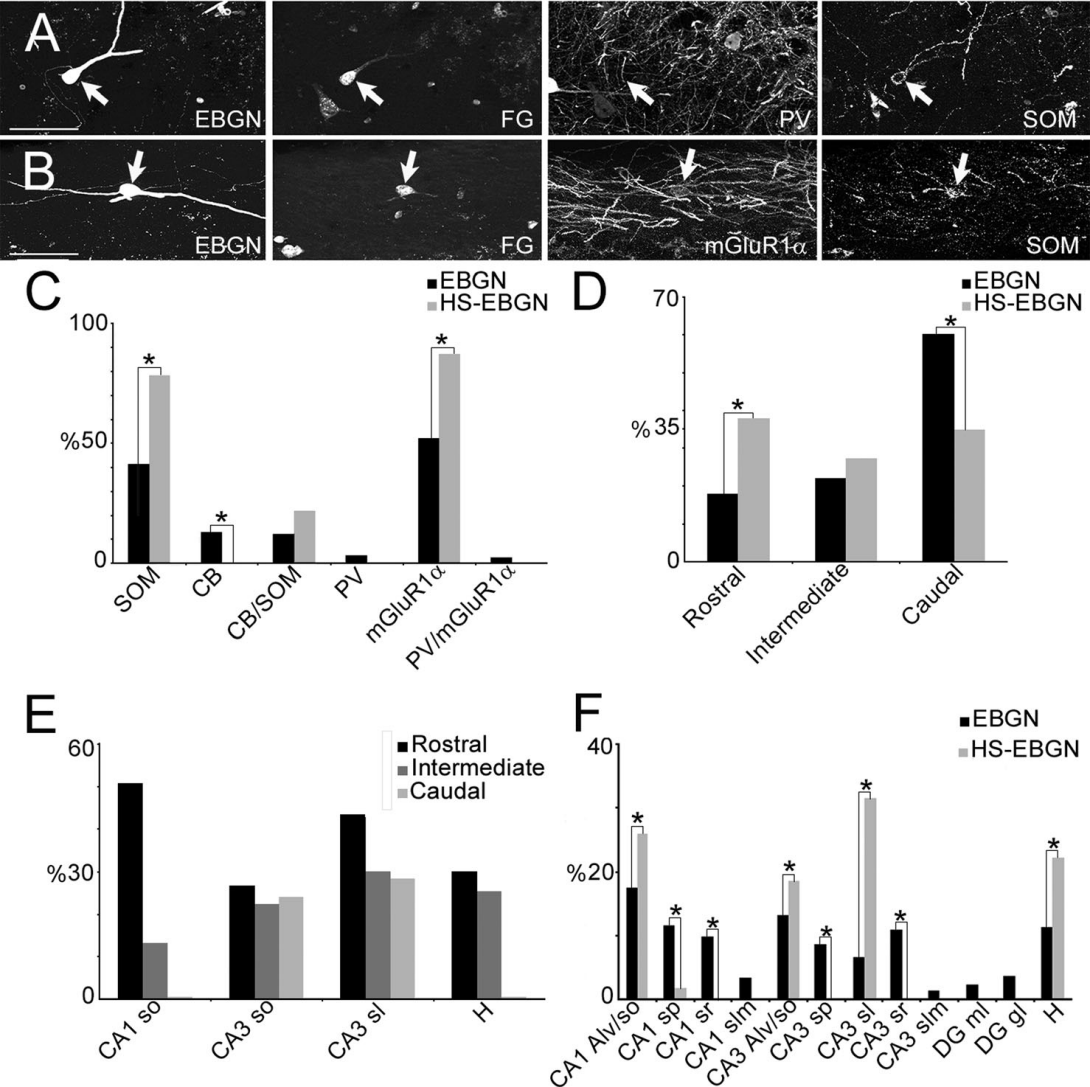
A subpopulation of EBGNs projects to the septum. **A,B:** Two retrogradely labeled EBGNs are in the stratum oriens of CA3 (arrows in A) and CA1 (arrows in B) in the hippocampus after injection of fluorogold (FG) in the septum. Both are immunopositive for somatostatin (SOM); one is also labeled for mGluR1α; one is negative for parvalbumin (PV). **C:** Histogram showing the fraction of hippocamposeptal EBGNs (HS‐EBGNs) immunoreactive for the various neurochemicals tested in comparison with the entire EBGN population. Note that 100% of HS‐EBGNs are positive for SOM. **D:** Histogram of the distribution of EBGNs and HS‐EBGNs along the rostrocaudal axis of the hippocampus. **E:** Histogram showing that the percentage of HS‐EBGNs among the EBGNs population decreases in hippocampal sublayers from the rostral to the caudal hippocampus; note that HS‐EBGNs represent half of the EBGNs in the rostral CA1 so. **F:** Histogram of the distribution of EBGNs and HS‐EBGNs in the various hippocampal areas and their sublayers. Alv, alveus; CB, calbindin; DG, dentate gyrus; gl, granular layer; H, hilus; ml, molecular layer; sl, stratum lucidum; slm, stratum lacunosum moleculare; sp, stratum pyramidale; so, stratum oriens; sr, stratum radiatum. *P < 0.05 as given by nonparametric bootstrap method. Scale bars = 50 μm.

In total 1715 EBGNs from 35 fluorogold‐injected adult mice were examined (Table 5). When fluorogold injections were applied to the medial septum, 17% ± 3% EBGNs were retrogradely labeled in the hippocampus (Fig. 7, Table 5). On the contrary, no EBGNs were labeled after injections in various regions of the neocortex, although many retrogradelly labeled neurons were present in hippocampus; a similar result was obtained after unilateral fluorogold injection in the hippocampus (Table 5).

**Table 5:** Target of Hippocampal Long‐Range Projecting Early‐Born GABA Neurons (EBGNs)

Fluorogold (FG) injection site	EBGNs	FG‐EBGNs	FG‐EBGNs (mean %)	Mice
CA1 contralateral	186	0	0	5
CA3 contralateral	108	0	0	5
DG contralateral	160	0	0	5
Dorsal subiculum retrosplenial cortex	323	0	0	4
Entorhinal cortex	251	0	0	6
Prelimbic area	325	0	0	5
Medial septum	362	54	16.7 ± 2.7	5

The expression of various neurochemical markers for GABA neurons, including parvalbumin (PV), somatostatin (SOM), calbindin (CB), and the metabotropic glutamate receptor mGlur1α, was tested and quantified for hippocamposeptal EBGNs (HS‐EBGNs) compared with those EBGNs that were not retrogradely labeled (Fig. 7A–C). In total 54 HS‐EBGNs and 303 EBGNs from for adult mice were analyzed for expression and regional distribution of neurochemical markers. All HS‐EBGNs were immunoreactive for SOM (Fig. 7; 23 tested cells); although the vast majority (87%, 31 tested cells) expressed mGluR1α, fewer expressed CB (22%, 23 tested cells), and none was positive for PV (31 tested cells). This is in agreement with previous studies analyzing the neurochemical content of hippocamposeptal projecting GABA neurons (Jinno and Kosaka, 2002; Jinno et al., 2007). However, it should be noted that the neurochemical signature of HS‐EBGNs differed from that of the total population of EBGNs. Indeed, a lower proportion of the overall EBGN population was positive for SOM, mGluR1α, or CB, with 61%, 59%, or 24% of colabeled EBGNs, respectively (Fig. 7C). Inversely, 5% of EBGNs were positive for PV. This indicates that HS‐EBGNs are likely to form a distinct subpopulation of EBGNs.

We next compared the distribution of HS‐EBGNs with that of all EBGNs throughout the rostrocaudal axis as well as within different hippocampal regions and layers (Fig. 7D,E). We observed that the rostrocaudal distribution of HS‐EBGNs was also different from that of the general population of EBGNs, with HS‐EBGNs preferentially distributed at the rostral level compared with the intermediate and caudal levels (Fig. 7D). Such difference in rostrocaudal distributions resulted largely from the significant preferential positioning of HS‐EBGNs in the CA1 stratum oriens at the rostral level because half of EBGNs at that particular location projected to the septum (Fig. 7E). EBGNs and HS‐EBGNSs were present throughout the hippocampus, with a higher proportion of HS‐EBGNs located in CA3 than in CA1 and in the dentate gyrus. Although EBGNs were distributed throughout all hippocampal layers, HS‐EBGNs were exclusively confined within the stratum oriens/alveus of CA1 (28%) and CA3 (18%), to the CA3 stratum lucidum (32%), and the hilus (22%; Fig. 7F). Therefore, EBGNs contain a subpopulation of GABA neurons projecting to the septum displaying a characteristic spatial distribution with a preferential location in the CA1 area of the dorsal hippocampus.

### Hippocampal EBGNs are targeted by extrahippocampal afferents in adult mice

We have shown that a significant fraction of EBGNs displays a long‐range extrahippocampal projection to the septum in the adult hippocampus, demonstrating that EBGNs connect hippocampus to other structures. In line with this result, we next tested the possibility that EBGNs are targeted by extrahippocampal afferents.

#### Septal afferents to hippocampal EBGNs

**Figure 8:**
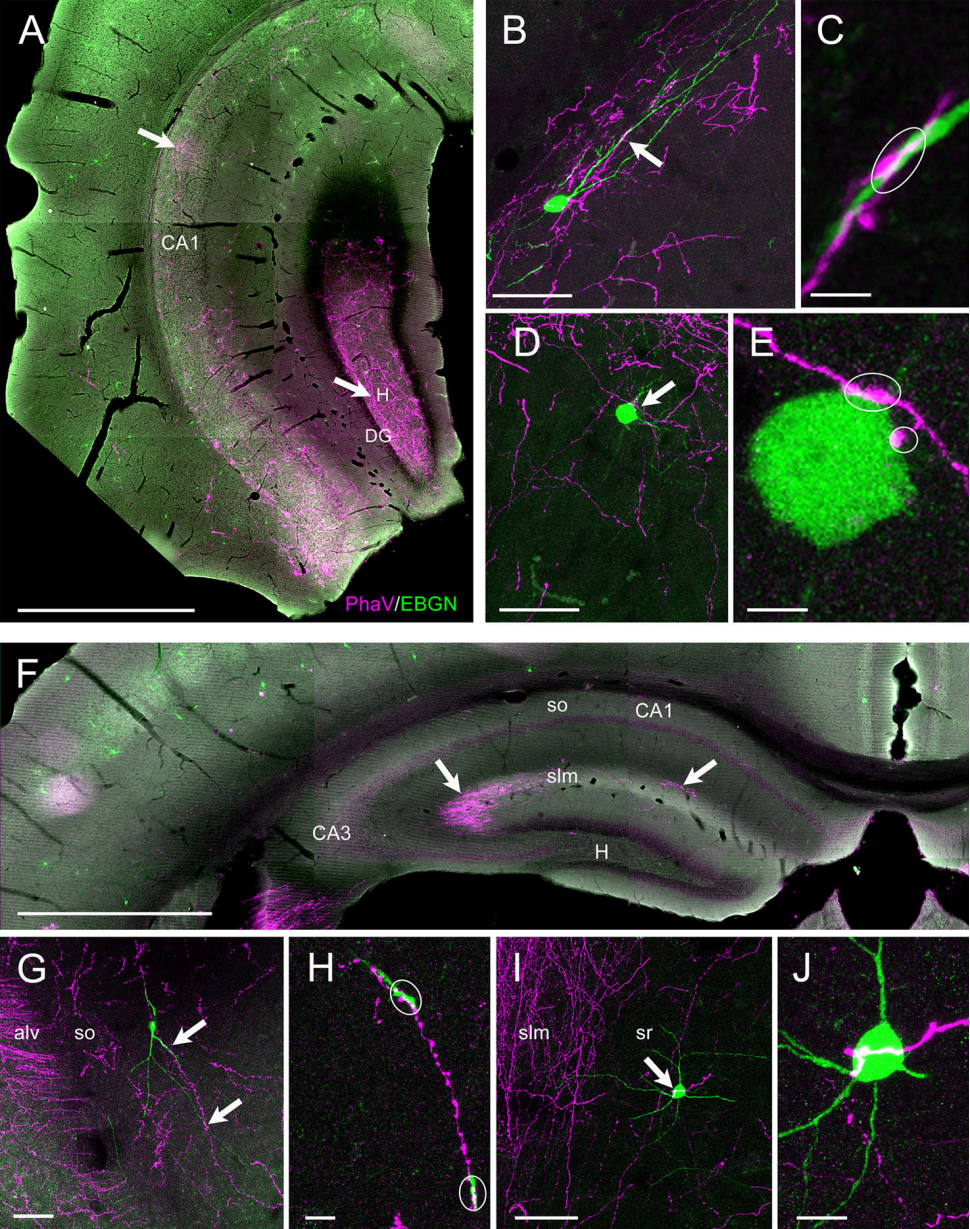
Septal and entorhinal afferents to EBGNs. **A–E:** Injection of *Phaseolus vulgaris* (PhaV) within the medial septum results in anterograde labeling of numerous fibers within the hippocampus. Numerous septal fibers are found in the hippocampus at the intermediate level in the stratum oriens of CA1 (arrows in A) and the hilus (H, arrows in A). B and D are confocal maximal‐intensity *z* stacks showing EBGNs in the stratum oriens of CA1 (B) and CA3 (D) targeted by anterogradely labeled septal fibers (arrows) on their soma and dendrites (outlined areas in C and E; C and E are high‐magnification single optical section from B and D z stacks, respectively). **F–J:** PhaV injection in the entorhinal cortex results in the anterograde labeling of numerous fibers within the stratum lacunosum moleculare (slm) of CA1 and CA3 (arrows in F); labeled fibers are also found in the stratum oriens (so) of CA1 (G). Confocal maximal‐intensity *z* stacks showing EBGNs in the stratum oriens of CA3 (arrows in G) and in the stratum radiatum of CA1 (arrow in I) targeted by entorhinal afferent fibers on dendrites and soma (outlined areas, H and J are high‐magnification single optical section from G and I z stacks, respectively). alv, Alveus; DG, dentate gyrus. Scale bars = 1 mm in A,F; 50 μm in B,D,G,I; 10 μm in H,J.

Septal injections of the anterograde tracer PhaV were restricted to the medial septum. The septal afferent labeling thus obtained was comparable to that in previous reports (Nyakas et al., 1987; Amaral and Lavenex, 2007). In the four adults that we examined after injections, multiple bulks of labeled fibers were visible all over the hippocampus but at different gradients of density (Fig. 8A). Fibers were more numerous in CA3 or CA1 strata oriens and CA3 stratum lucidum than in other hippocampal layers; they were also dense in the fimbria. We could observe two types of fibers, varicose thin fibers and thicker fibers (Fig. 8B–E). Contacts between PhaV‐labeled septal afferent fibers and EBGNs (Fig. 8B–E) were present in all injected animals and were easy to find despite the relatively restrictive injection parameters (small injected volume, single injection). Contacts were found on the soma and dendrites of EBGNs distributed throughout the hippocampus (Fig. 8B–E). We found that 25% of EBGN somata (27 of 108 EBGNs) were contacted by septal afferents. These results suggest that EBGNs are frequent recipients of septal afferents.

#### Perforant path afferents to hippocampal EBGNs

Injections of the anterograde tracer PhaV were restricted to the medial and lateral entorhinal cortices (four adult mice). In the cases when PhaV labeling diffused beyond the central injection site toward the perirhinal cortex, part of the amygdala, and the ventral part of CA1, animals were discarded. In agreement with the labeling of the perforant path (Deller et al., 1996; Amaral and Lavenex, 2007), most of the PhaV‐labeled entorhinal afferent fibers were observed in the statum lacunosum moleculare of CA3 and CA1 and to a lesser extent in the molecular layer of the dentate gyrus (Fig. 8F). Fewer fibers of entorhinal origin could be observed in the stratum oriens of CA1 and CA3 corresponding to alvear pathway labeling (Fig. 8G; Deller et al., 1996). Contacts between entorhinal afferents and EBGNs were sparse. They were found on the soma or dendrites of hippocampal EBGNs in the CA1 and CA3 in the stratum lacunosum moleculare and also in the stratum oriens (Fig. 8G,H) and the stratum radiatum (Fig. 8I,J). In conclusion, we have shown that hippocampal EBGNs could receive inputs originating from the medial and/or lateral entorhinal cortex.

#### Cholinergic and monoaminergic afferents to hippocampal EBGNs

**Figure 9:**
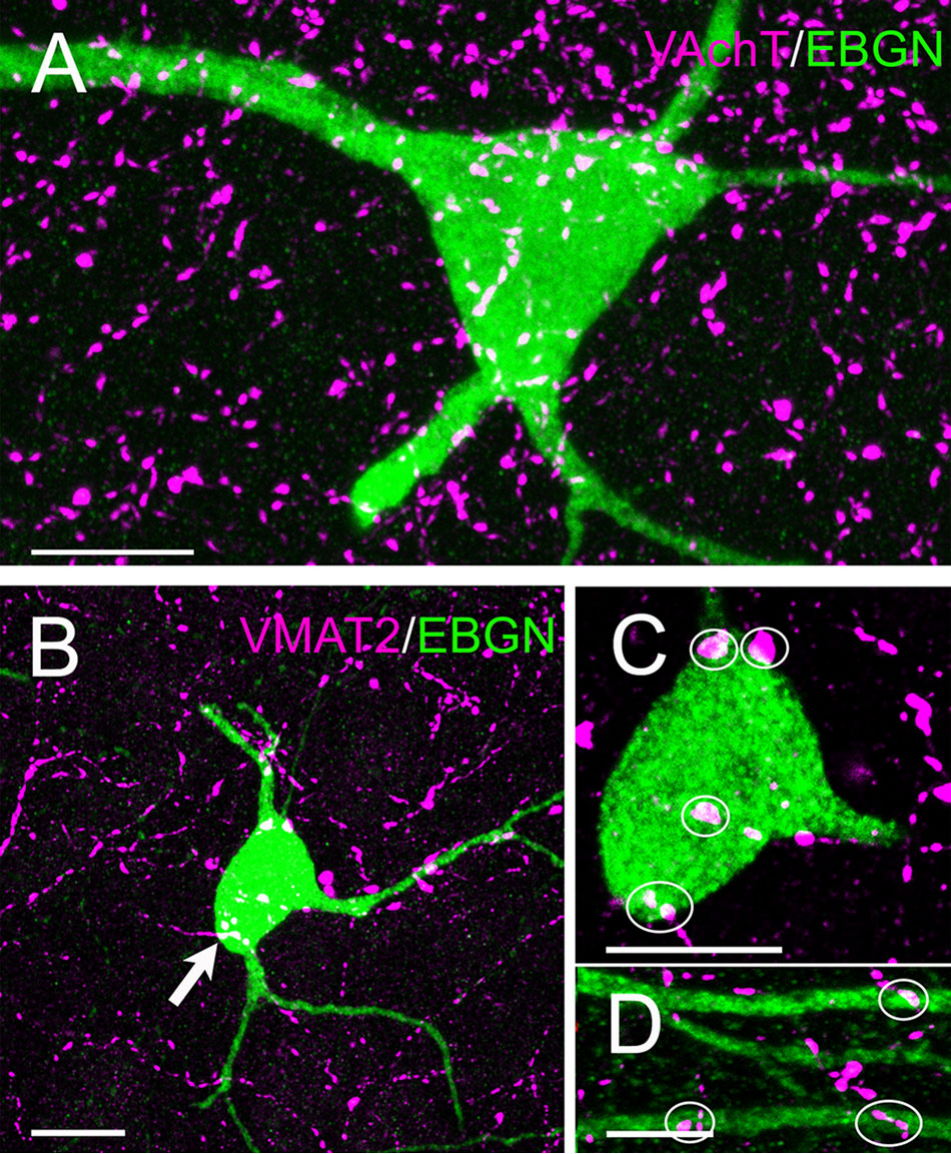
Subcortical afferents to EBGNs. **A:** EBGNs are covered by cholinergic fibers labeled for the vesicular acetylcholine transporter (VAchT). **B–D:** EBGNs are contacted by monoaminergic fibers (vesicular monoamine transporter VMAT2) on their soma (outlined areas, C) and dendrites (outlined areas, D). Scale bars = 10 μm in A–C; 5 μm in D.

We have demonstrated that some EBGNs display a long‐range projection to the septum and that in turn they receive afferents from the perforant path and septum. It is well known that hippocampal neurons are also contacted by monoaminergic afferents originating from distant areas that include mainly the median raphe nucleus, the ventral tegmental area (VTA), the substantia nigra, and the locus coeruleus (Foote et al., 1983; Freund et al., 1990; Gasbarri et al., 1994a, 1994b; Varga et al., 2009; Walling et al., 2012). In addition, hippocampal neurons are also the site of long‐distance cholinergic inputs originating from the medial septum and the nucleus of the diagonal band (Amaral and Lavenex, 2007; Teles‐Grilo Ruivo and Mellor, 2013). Monoaminergic afferents were visualized by labeling for VMAT2. VMAT2 is the vesicular transporter for neurons synthesizing and storing dopamine, serotonin, adrenaline, and noradrenaline (for review see Eiden and Weihe, 2011). Cholinergic inputs were labeled with VAchT, and we found that all the EBGNs observed received strong inputs from both cholinergic (40 EBGNs from three adult mice; Fig. 9A) and monoaminergic (39 EBGNs from three adult mice; Fig. 9B–D) inputs.

### EBGNs from different structures display long‐range GABAergic projections in adult mice

**Figure 10:**
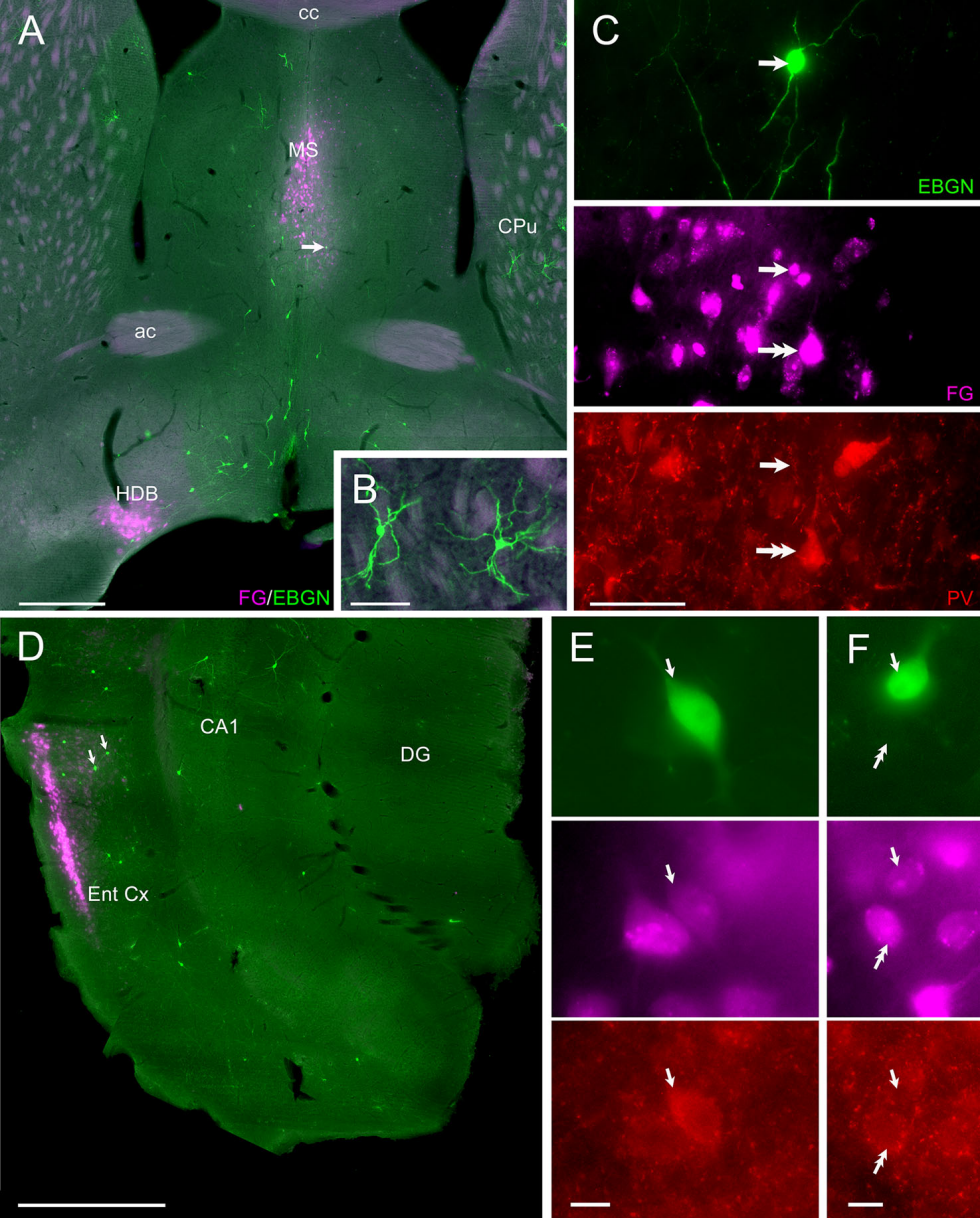
EBGNs form a subpopulation of GABA long‐range projecting neurons. **A–C:** Numerous EBGNs are observed in the medial septum (MS) and in the horizontal diagonal band (HDB). One of them (single arrows in A,C) is retrogradely labeled after hippocampal fluorogold injection (FG). It is not positive for parvalbumin (PV; single arrows in C), although other retrogradely labeled neurons are positive (double arrow in C). Note the presence of EBGN medium spiny neurons in the caudate putamen (CPu, in A,B). **D–F:** EBGNs are present in the entorhinal cortex (Ent Cx); two of them (single arrows in D–F) are retrogradely labeled; one is positive for PV (arrows in E). Note the presence of a neighboring PV retrogradely neuron (double arrows in F). ac, Anterior commissure; cc, corpus callosum; DG, dentate gyrus. Scale bars = 500 μm in A,D, 100 μm in B,C; 10 μm in E,F.

Fate mapping of neuron precursors expressing *Dlx1/2* at early embryonic stages (<E10) resulted in the labeling of a subpopulation of EBGNs in different areas of the forebrain, including the neocortex, the hippocampus, the striatum, the septum, and the amygdala. Very occasional EBGNs were observed in the other structures of the forebrain. EBGNs were found throughout the neocortical layers. In the striatum, EBGNs could be detected and presented the morphological features of medium spiny neurons (Fig. 10A,B). Some EBGNs were visible in the different subnuclei of the amygdala. EBGNs were also present in the septum (Fig. 10A,C). Given that significant numbers of hippocampal EBGNs project outside the hippocampus and that striatal EBGNs include medium spiny neurons, which are also well known to be GABAergic projection neurons, we finally asked whether such long‐range GABAergic projection is a common property for EBGNs from different cortical regions. We focused on the septum and entorhinal cortex because we observed that EBGNs could be found in both regions and because both regions had been reported to display a GABAergic projection to the hippocampus (Freund and Antal, 1988; Melzer et al., 2012). We thus tested for the presence of retrogradely labeled septal or cortical EBGNs after bilateral fluorogold injections in the dorsal hippocampus of five adult mice (Table 1).

EBGNs were present along the rostrocaudal axis of the septum (Fig. 10A). Bipolar smooth neurons were seen in the medial septal nucleus and the nuclei of the vertical or horizontal limb of the diagonal band. In addition, some rare multipolar and spiny neurons were observed in the lateral septum. Given that septal neurons projecting to the hippocampus include PV‐containing cells (Borhegyi et al., 2004), septal EBGNs (two animals, 53 cells) were tested for PV expression. but none was positive for PV (Fig. 10C). However, retrograde labeling could be observed in a small fraction of septal EBGNs (3% ± 2% of the total septal EGBNs population, 19 of 644 EBGNs; Fig. 10A,C), thus indicating that septal EBGNs include cells that project from the septum to the hippocampus.

In the entorhinal cortex, EBGNs were relatively rare and were distributed mostly in deeper layers (Fig. 10D–F). Still, we were able to find some rare entorhinal EBGNs that were retrogradely labeled after fluorogold injections in the hippocampus (2% ± 2% of the total entorhinal EBGN population; 5/229 neurons; Fig. 10E,F). Therefore, entorhinal EBGNs also include cells that project outside from the entorhinal cortex to the hippocampus, further supporting the possibility that EBGNs in different brain areas are preferentially GABAergic projection neurons.

**Figure 11:**
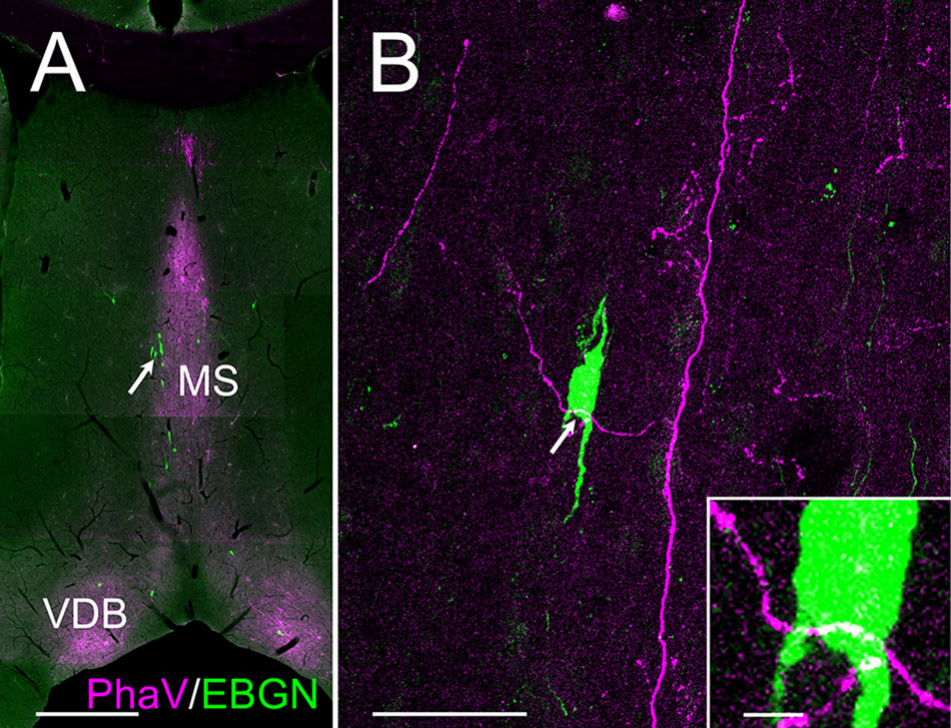
Hippocampal afferents to septal EBGNs. **A:** PhaV injection in CA1 of dorsal hippocampus results in the presence of labeled fibers within the medial septum (MS) and the ventral diagonal band nucleus (VDB) beside septal EBGNs (arrow in A). **B:** One hippocampal fiber contacts a septum EBGN (arrow in B and **inset**). Scale bars = 500 μm in A; 50 μm in B; 5 μm in inset.

We found that EBGNs were involved in the septohippocampal loop, so we tested the possibility that some hippocampal EBGNs could also in turn contact septal EBGNs. With this aim, we injected PhaV bilaterally within the dorsal CA1, hoping for the colabeling of EGFP and PhaV within axons of EBGNs targeting the septum (five adult mice; Table 1). Unfortunately, for unexplained reasons, the hippocampal EBGNs could not be labeled by PhaV, although other cells (pyramidal cells, putative interneurons) were labeled. Accordingly, we could never observe PhaV/EGFP double‐labeled fibers within the septum. Nevertheless, numerous anterogradely labeled hippocamposeptal fibers were observed in the medial septum (Fig. 11A). Few of them contacted septal EBGNs (Fig. 11B). Therefore, septal EBGNs receive hippocampal inputs, but we are not able to determine whether some of these inputs originate from hippocampal EBGNs.

## DISCUSSION

Using a combined full description of morphological and electrophysiological parameters paralleled with imaging of early network dynamics, we show that the development of the GABA neurons that are generated earliest and operate as hub cells mirrors the emergence of coordinated synapse‐driven activities and later provides a long‐range GABAergic link between the hippocampus and the septum. This study provides a detailed template for future studies aiming at the analysis of hub neuron dysfunctional development, a condition that may well relate to developmental brain pathologies given the importance of GABA neurons and activity‐dependent mechanisms in the latter.

### The maturation of early‐born hub cells parallels the emergence of synapse‐driven network patterns

At early postnatal stages, GABA neurons display heterogeneous stages of development (Gaiarsa et al., 2001; Hennou et al., 2002). We have recently shown that the morphophysiological features of GABA neurons develop in accordance with their internal developmental time schedule, which is largely set by their date of birth (Allene et al., 2012). In agreement with these observations, we report an early development of EBGNs. Several classical electrophysiological parameters have served here as indirect indicators of cellular growth, such as an increase in membrane capacitance, a decrease in input resistance, or a maturation of action potential properties with a lower threshold and faster kinetics. Previous authors have reported comparable evolutions of intrinsic electrophysiological features in cortical or subcortical structures (Okaty et al., 2009; Matta et al., 2013). At P7, the EBGNs intrinsic electrophysiological properties were similar to those recorded in juvenile hippocampus (P14–P21) and in adult animals (present data), suggesting an advanced maturation (Cossart et al., 2006). Nevertheless, the action potential kinetics of many interneurons and in particular basket cells continue maturing until advanced postnatal stages (P25; Doischer et al., 2008). In addition, EBGNs, which are recruited early into GDPs, progressively integrate into functional synaptic microcircuits as the rate of the sEPSPs increases by almost fourfold during the first postnatal week, most likely through the development of glutamatergic synapses. Indeed, EBGNs probably receive inhibitory rather than excitatory GABAergic inputs, as suggested by their early expression of the chloride extruder KCC2 at the membrane. The diverse firing patterns displayed by EBGNs, including regular spiking, are also indicative of an earlier maturation in comparison with later‐born neurons (Allene et al., 2012).

This early development is not a smooth, continuous process because we observed an acceleration of the evolution of this combination of indicators at about P3 (Fig. 5). At the network level, this is the time when the SPA to GDP replacement process begins in CA3, as shown here. In CA1, the emergence of correlated neuronal activities, similar to the maturation of cellular properties, also followed a precise schedule, with the earliest coherent electrical pattern of activity arising about at birth (but P3 here in CA3) in the form of SPAs being progressively replaced by GDPs toward the end of the first postnatal week (Crepel et al., 2007). We describe a similar phenomenon in CA3, as expected from the fact that such sequence has been reported in other structures, including the neocortex (Allene et al., 2008) and even the striatum (Dehorter et al., 2011). It will be important to understand what could be occurring at P3 that accelerates the development of EBGNs and GDPs and in particular whether both phenomena are causally related, as reported for the glutamatergic innervation of basket cells that terminates GDPs (Pelkey et al., 2015).

The neurochemical markers expressed by EBGNs indicate a dendritic, rather somatic innervation preference (strong SOM vs. minor PV expression). The development of dendrite‐projecting GABA neurons opens the time for GDPs dominating network dynamics whereas the integration of somatic‐projecting ones into functional glutamatergic circuits closes that period (Pelkey et al., 2015). Whether there is a specific signaling between these two microcircuits that triggers the end of synapse‐driven synchronizations, as happens in other structures such as the retina (Blankenship et al., 2009; Blankenship and Feller, 2010), remains to be determined.

The selective invalidation or silencing of EBGNs at specific times will address this issue but is experimentally challenging because EBGNs are available to genetic manipulation only at very early embryonic time points. In any case, these few perinatal days represent a susceptibility time frame for environmental insults or genetic disorders to affect hippocampal maturation.

### A pioneer network of cortical GABA dendrite‐projecting neurons with long‐distance connections

We had previously shown that EBGNs remained into adulthood, when they expressed a combination of neurochemical markers identifying them as putative long‐range projecting GABA neurons (Picardo et al., 2011). However, the exact extrahippocampal projection pattern of early‐born GABAergic hub neurons had to be determined because GABAergic projection neurons have been reported to target a variety of structures, including the entorhinal cortex, subiculum, retrosplenial cortex, septum, and amygdala (Ceranik et al., 1997; Losonczy et al., 2002; Ferraguti et al., 2005; Jinno et al., 2007; Fuentealba et al., 2008; Jinno, 2009; Melzer et al., 2012). The combination of anterograde and retrograde labeling performed here in adult animals allowed identifying the septum as the main target area of EBGNs, with almost half of the labeled EBGNs in the dorsal CA1 targeting this structure. Our results support the idea that EBGNs participate to hippocamposeptal connections not only in adulthood but also during development. Indeed, the axons of some of them were found running within the fimbria as early as E18.5. This is in agreement with the early development of a pioneer GABAergic pathway from the rat hippocampus, reaching the septum as early as E16, prior to glutamatergic afferents and septohippocampal connections (Linke et al., 1995). It is also in agreement with the observation that GDPs recorded in the septum in the neonatal intact septohippocampal complex in vitro, originate in the dorsal hippocampus and propagate to the septum via hippocamposeptal projecting neurons (Leinekugel et al., 1998). Furthermore, hippocamposeptal somatostatin neurons have been shown to synchronize pathological network activity in the CA1 region of immature hippocampus (Quilichini et al., 2012). This population may include EBGNs given that all hippocamposeptal EBGNs in CA1 express somatostatin. Therefore, it can be suggested that EBGNs are acting as operational hub neurons in CA1, at least under pathological conditions. In adulthood, hippocampal long‐range projecting somatostatin neurons are also involved in synchronous activity because they strongly increase their firing rate during sharp wave ripples (Jinno et al., 2007). In parallel with their projection to the septum, EBGNs are, in turn, frequent recipients of septal afferents. Moreover, EBGNs are densely innervated by cholinergic inputs that within the hippocampus originate mainly from the medial septum/diagonal band of Broca complex (for review see Teles‐Grilo and Mellor, 2013; Alger et al., 2014). In addition, part of them was found to express muscarinic type 2 receptors (M2R; Picardo et al., 2011). Altogether these results support the idea that hippocampal EBGNs are a subpopulation of hub neurons that may play a crucial role in the hippocamposeptal system during development (Quilichini et al., 2012) or later (Jinno et al., 2007; Fuhrmann et al., 2015). This may place EBGNs in a critical position for setting synchronous activity within the hippocampus–septum system through cholinergic septohippocampal afferents (Vandecasteele et al., 2014) and/or reciprocal hippocamposeptal GABAergic connections (Takács et al., 2008).

Hippocampal EBGNs are frequently contacted by monoaminergic fibers in adult mice as demonstrated by our VMAT2 labeling, suggesting that their activity may be modulated by monoamines. Thus, serotonin may modulate the activity of the subpopulation of EBGNs, as observed for GABA neurons (Varga et al., 2009; Winterer et al., 2011). Noradrenergic receptor agonists can excite, inhibit, or have no influence on various subtypes of GABA neurons throughout the different layers of the CA1 region (Parra et al., 1998; Zsiros and Maccaferri, 2008); almost all CA1–CA3 stratum oriens somatostatin neuron express the β1‐adrenergic receptor (Cox et al., 2008), and CA1 stratum oriens GABA neurons have been shown to express α1‐adrenergic receptors (Papay et al., 2006). From this we can conclude that adrenergic and/or noradrenergic afferents may affect EBGN activities and probably the activity of septum‐projecting ones. A similar conclusion can be drawn for dopaminergic afferents because the dopamine D2 receptor has been detected in a subclass of GABA neurons expressing mGluR1α and/or somatostatin and located in the stratum oriens of CA1 and CA3 of the dorsal hippocampus, corresponding to the neurochemical profile and location of HS‐EBGNs (Puighermanal et al., 2015).

During development, EBGNs have been demonstrated to be operational hub neurons displaying a high degree of functional connectivity but also actively involved in generating network synchronization, the stimulation of which single‐handedly impacts network dynamics (Picardo et al., 2011). According to their anatomical features, EBGNs within the adult hippocampus present the characteristics of both connector hubs with a long axon running through the fimbria to the septum and provincial hubs with a dense local arborization (Cossart, 2014). Indeed, GABAergic neurons projecting to the septum have been shown to target locally within the hippocampus both interneurons and pyramidal cells (Takacs et al., 2008). We have demonstrated that, in addition to their local and/or long‐range axonal arborization, hippocampal EBGNs are the recipients of various extrahippocampal afferents arising from the septum/diagonal band of Broca complex, perforant path, midbrain, and pons structures. Thus, adult EBGNs could play a role in determining in the communication between the hippocampus and other cerebral structures.

The fact that some EBGNs develop into a population of GABA neurons with a long‐range projection may represent a general developmental feature not restricted to hippocampal cells. Indeed, we observed here in rare cases retrograde labeling of EBGNs in the septum and entorhinal cortex following hippocampal injections. Small numbers are a problem when considering experiments with mild success probability, such as the long‐range tracing experiments performed here. Hence, the probability of double retrograde and EGFP labeling is so low that the repeated observation of double‐labeled neurons most likely identifies a robust property of EGBNs. These EBGNs likely constitute a less well‐documented subtype of GABA projection neurons, because PV colabeling was not observed in the septum and only very occasionally in entorhinal cortex; indeed, PV is the neurochemical content most frequently associated with these cells in the septum (Freund, 1989; Hangya et al., 2009) and entorhinal cortex (Melzer et al., 2012). Moreover, the capability of EBGNs to develop into a population of GABA neurons with a long‐range projection may not be restricted to cortical structures, as exemplified by the presence of medium spiny neurons in the striatum. Future studies are needed to determine whether EBGNs also connect to one another following a rich club topology (Schroeter et al., 2015) and/or a temporally matched connectivity rule (Donato et al., 2015), thus forming an early developmental scaffold upon which adult topology and dynamics build.

## CONFLICT OF INTEREST STATEMENT

The authors declare they have no known or potential conflicts of interest.

## ROLE OF AUTHORS

VV, PG, and MAP contributed equally to this work. Study concept and design: VV, PG, MAP, RC, AB. Acquisition of data: VV, PP, MAP, PL, AB. Analysis and interpretation of data: VV, PG, MAP, RC, AB. Drafting of the manuscript: RC, AB. Statistical analysis: AM Obtained funding: RC. Technical and material support: TT, VS, EL.
